# Surfactant-treated graphene covered polyaniline nanowires for supercapacitor electrode

**DOI:** 10.1186/s11671-015-0888-1

**Published:** 2015-04-16

**Authors:** Balasubramaniyan Rajagopalan, Seung Hyun Hur, Jin Suk Chung

**Affiliations:** School of Chemical Engineering, University of Ulsan, 93 Daehakro, Namgu, Ulsan, 680-749 Republic of Korea

**Keywords:** PANI nanowire, Graphene, Nanocomposites, Supercapacitor

## Abstract

Surfactant-treated graphene/polyaniline (G/PANI) nanocomposites were prepared by the MnO_2_ template-aided oxidative polymerization of aniline (ANI) on the surfactant-treated graphene sheets. The electrochemical performances of the G/PANI nanocomposites in a three-electrode system using an aqueous sulfuric acid as an electrolyte exhibited a specific capacitance of 436 F g^−1^ at 1 A g^−1^, which is much higher than the specific capacitance of pure PANI (367 F g^−1^). Such a higher specific capacitance of the G/PANI nanocomposite inferred an excellent synergistic effect of respective pseudocapacitance and electrical double-layer capacitance of PANI and graphene.

## Background

To develop an advanced supercapacitor, low cost, eco-benignity, and high efficiency are indispensable. Based on these needs, much interest has been focused on the electrode materials for supercapacitor to improve both the power and energy density [1-3]. Recently, high surface area carbons, metal oxides, and conducting polymers are frequently used as the advanced material in supercapacitor [4]. Two-dimensional (2D) graphene is widely used for energy storage devices owing to its outstanding properties [5]. The preparation of porous graphene with a high surface area by the introduction of pseudocapacitive materials such as metal oxides or conducting polymers to graphene sheets for hybrid materials is one of the efficient approaches for improving the performance of graphene-based supercapacitors [6,7].

Conducting polymers such as polyaniline (PANI), polypyrrole, and polythiophene have been extensively studied for pseudocapacitive materials [8]. Higher energy storage capacitances result owing to the fast faradaic reactions between the electrode materials with unique proton doping and dedoping mechanism of conducting polymers [9]. The drawbacks of these pseudocapacitive materials are the lack of long-term stability and rather slow charge/discharge time because of the swelling and shrinking of polymers [10]. The polymers of definite arrangement and well-conductive path for ion and electron transport are crucial to develop PANI with superior electrochemical performances. The polymer particles between the conducting materials (graphene or CNT) efficiently provide a higher electroactive region and increase the effective access of the electrode in both the double layer and redox mechanism [8,10,11]. The 2D graphene is a good choice for polymer nanocomposites owing to its excellent electrical and mechanical properties and increased electrochemical performances.

The OH, COOH, and epoxy functional groups of graphene oxide (GO) easily interact with polymer backbones to improve the properties of the nanocomposites [4,11-13]. However, the higher electrical resistance of GO or GO with polymer nanocomposites results in poor electrochemical performances in supercapacitor electrode. In contrast, reduced GO is a good choice for double-layer capacitors, as it improves the electrical conductivity of the nanocomposites [14]. The aggregation of graphene nanosheets is an obstacle in the application of the reduced GO in solution, hindering the electrochemical performances [15,16]. The functionalization or surfactants have been used for preventing the aggregation of the graphene sheets [17,18]. A uniform morphology of hybrid material is also essential for the successful electrolyte transport on the electrode-electrolyte boundaries [1,19]. Based on these strategies, this study is focused on the improvement of the energy storage performance of PANI via the intercalation of PANI and surfactant-treated graphene sheets for supercapacitor electrode. Recently, aggregation-free graphene nanocomposites were obtained via the encapsulation of the graphene sheets on the surface of PANI or metal oxides [20,21]. The wrapping of the graphene sheets is helpful for supercapacitor, which significantly increases the charge transport characteristics of the PANI or metal oxide electrodes.

Herein, we describe the preparation of graphene/polyaniline (G/PANI) nanocomposites by inserting MnO_2_ between the surfactant-treated graphene sheets and followed by oxidative polymerization of aniline (ANI). Sodium lauryl sulfate (SLS) used as the surfactant prevents the aggregation of graphene sheets and spacer for accommodating the PANI between the graphene sheets. By varying the ratio of graphene and PANI, the effect of specific capacitance was studied.

## Methods

### Materials

Expandable graphite (Grade 1721) was obtained from Asbury Carbon (Asbury, NJ, USA). Concentrated sulfuric acid (H_2_SO_4_), potassium permanganate (KMnO_4_), hydrochloric acid (HCl), hydrogen peroxide (H_2_O_2_), and manganese (II) sulfate monohydrate were received from Samchun Chemical Co., Ltd. (Gyeonggy-do, Korea). Hydrazine monohydrate (N_2_H_4_-H_2_O), potassium dichromate, SLS, and ANI were provided by Sigma-Aldrich (St. Louis, MO, USA). All the chemicals were used as received without further purification.

### Preparation of GO-SLS/PANI nanocomposite

MnO_2_ was prepared following the literature procedure [22], and GO was prepared according to the modified Hummers method [23]. GO (200 mg) was suspended in DI water for approximately 2 mg/mL. SLS (1.2 g) was added to the GO suspension, while stirring and sonication for 30 min. A predetermined quantity of MnO_2_ was added, and the stirring was continued for an additional 30 min. The GO-SLS/MnO_2_ solution was taken in a 500-mL beaker and placed in an ice-cold water. While stirring, ANI dispersed in DI water and HCl (2 M) were added, and the reaction was continued for approximately 3 h. A viscous black-colored solution of GO-SLS/PANI was obtained at the end of the reaction.

### Preparation of G/PANI nanocomposites

The viscous solution of GO-SLS/PANI was placed in an oil bath, and the temperature was set to 90 C. Hydrazine hydrate (1:1 with respect to GO) was added to that solution, and the reaction was continued for 1 h. The reduced GO-SLS/PANI was washed with 1 M HCl and ethanol to completely remove SLS from the nanocomposite solution. Finally, the G/PANI nanocomposites were dried at 60 C for 24 h to obtain the nanocomposite powders. The blank graphene sample was also prepared by the reduction of the surfactant-treated GO without using PANI. The G/PANI nanocomposites were labeled as G/PANI (GP) 46 and GP 64 according to the weight percent of graphene in the final product.

### Electrode preparation and electrochemical measurements

All the electrochemical measurements were performed in a three-electrode cell assembly equipped with a reference electrode (Ag/AgCl), a platinum wire counter electrode, and a glassy carbon working electrode. The electrode was fabricated by sonication in 85% of active material, 10% super-P carbon black, and 5% polytetrafluoroethylene in 2 mL of ethanol solution. The electrochemical performances were investigated using a 2 M aqueous sulfuric acid as the electrolyte. The mass of active material for the electrochemical measurement was about 1.5 mg. The electrochemical charge/discharge and cyclic voltammetry (CV) were performed in the potential window range of −0.2 to 0.8 V. The electrochemical impedance spectra (EIS) were measured at frequencies between 100 kHz and 0.01 Hz.

### Instruments

Morphologies of the G/PANI nanocomposites were characterized via field-emission scanning electron microscopy (FE-SEM, JSM-6500 F, JEOL Ltd., Akishima-shi, Japan). Thermal properties were characterized using a thermogravimetric analysis (TGA, Q50, TA Instruments, New Castle, DE, USA). Functional group of the nanocomposites was determined using a Nicolet IR 200 FTIR spectrometer (Thermo Scientific, Waltham, MA, USA) and the *I*_*D*_ and *I*_*G*_ values of the graphene were determined using a Raman (DXR; Thermo Scientific, Waltham, MA, USA). The charge/discharge and CV of the G/PANI electrode were performed by using a battery tester (Won A Tech, WBCS 3000, Seoul, Korea). EIS was measured using Bio Logic Science Instruments (Claix, France).

## Results and discussion

**Figure 1:**
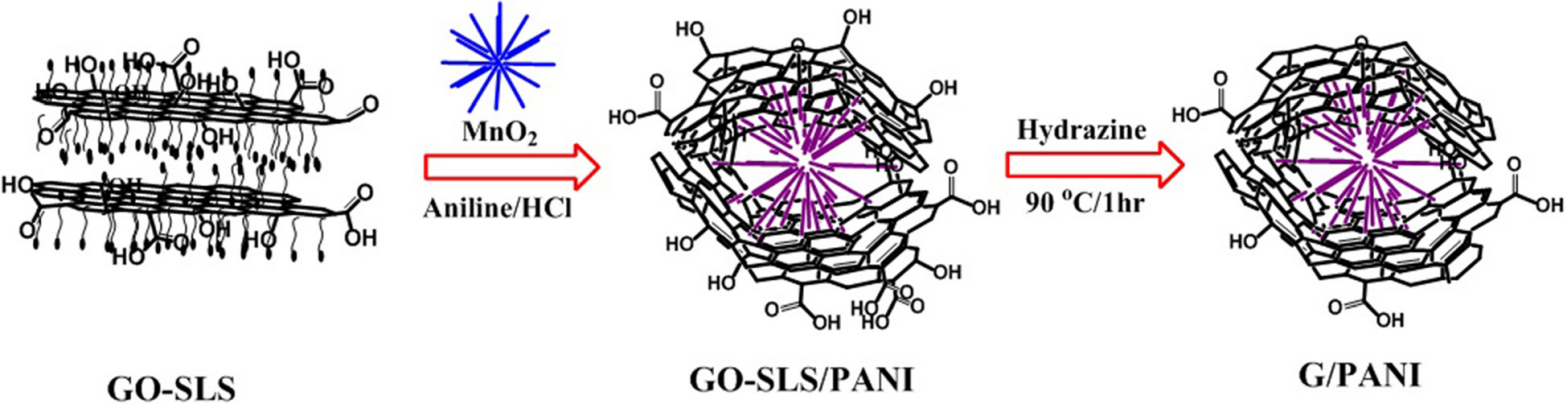
Preparation of G/PANI nanocomposites. Systematic representation for the intercalation of PANI nanowires and G.

**Figure 2:**
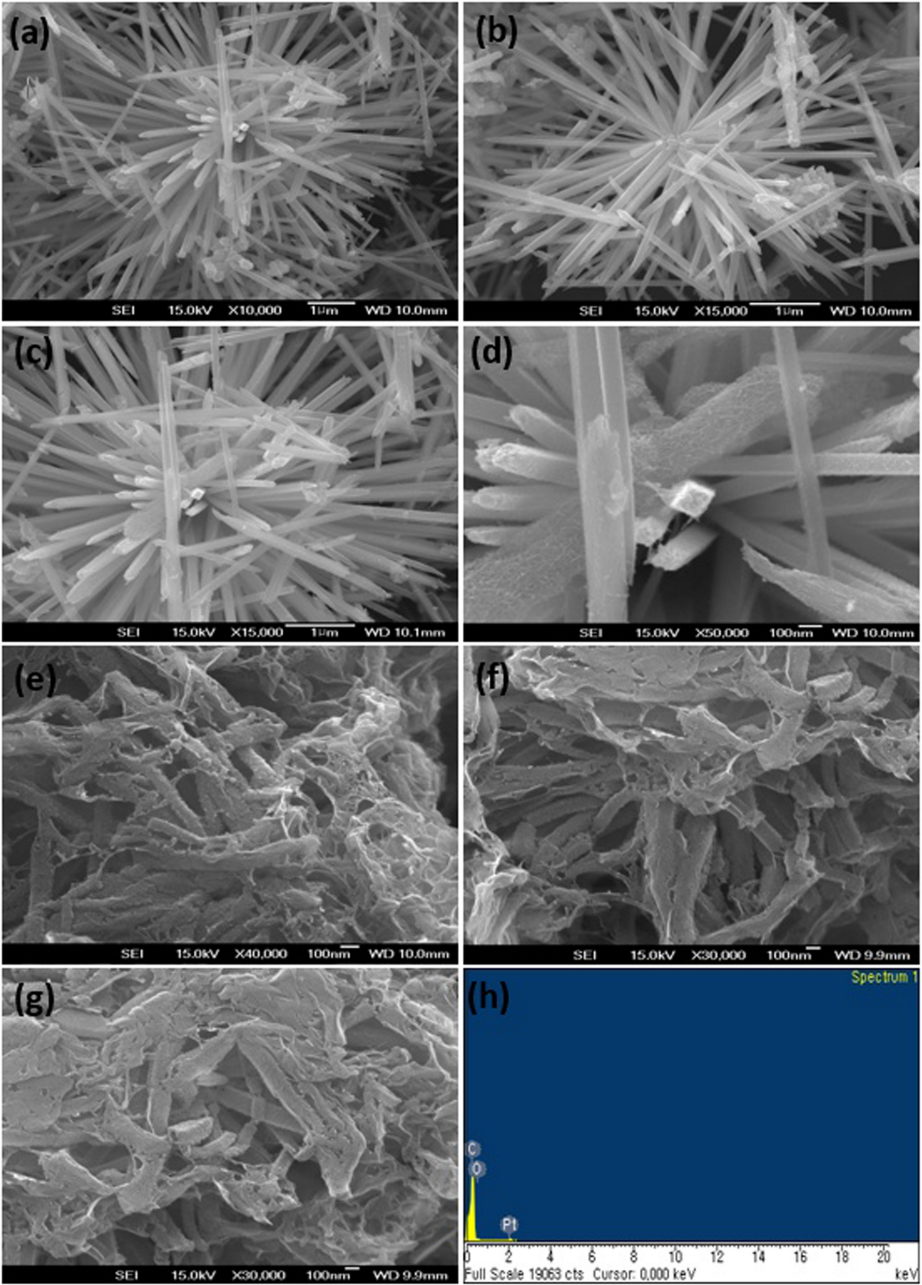
FE-SEM images of the nanocomposites. FE-SEM images of MnO_2_ nanowires **(a-d)**, G/PANI nanocomposites **(e-g)**, and EDAX of the G/PANI nanocomposite **(h)**.

The template-aided preparation of G/PANI nanocomposites was aimed at reducing the aggregation of the nanocomposites, which improves the electrical conductivity and provides the sufficient electrolyte accessibility to the electrode/electrolyte interface. The layer stacking and non-uniform deposition of the PANI on the G sheets can be greatly avoided. The synthesis scheme for the G/PANI nanocomposite is shown in Figure 1. The intercalation of MnO_2_ on the surfactant-treated graphene significantly prevents the aggregation of the nanocomposites. The MnO_2_ obtained by the hydrothermal method produces a nanowire of a needle-like morphology (Figure 2). Figure 2a,b,c,d shows the nanowire structure of MnO_2_. The sizes of nanowires varied between 2.6 and 3.5 μm, and their lengths and width varied between 121 and 130 nm. Figure 2e,f,g shows the morphology of the G/PANI nanocomposites. The PANI formed by the oxidative polymerization is almost similar in morphology to those of the MnO_2_ nanowires. Figure 2e,f,g clearly shows the intercalated morphologies of PANI and graphene sheets. Because of the electrostatic interactions between the graphene and PANI, the graphene sheets covered the entire area of the PANI nanowires. The detailed mechanism for the formation of PANI nanowires from the MnO_2_ template is given below [22]:

$$ \mathrm{A}\mathrm{N}\mathrm{I}+{\mathrm{Mn}\mathrm{O}}_2+4{\mathrm{H}}^{+}+2{\mathrm{e}}^{\hbox{-}}\to {\mathrm{Mn}}^{2+}+2{\mathrm{H}}_2\mathrm{O}+\mathrm{PANI} $$

In the presence of strong acid, ANI polymerization occurred at the surface of the MnO_2_ nanowires and simultaneously degraded the Mn to soluble Mn^2+^ ions, then ANI replaced the MnO_2_, and finally formed PANI nanowires [22]. While oxidative polymerization of ANI, some of ANI monomers were polymerized on the graphene surfaces because of the doping via the *π*-*π* interactions of ANI and GO. The coating of PANI on the graphene sheets increases the contact area of the electrode, improving the electrochemical performance of the G/PANI nanocomposites [7]. The absence of Mn peak in the energy dispersive X-ray analysis (EDAX) spectrum of the G/PANI nanocomposites strongly proved the complete removal of Mn as soluble Mn^2+^ ions in solution (Figure 2h).

**Figure 3:**
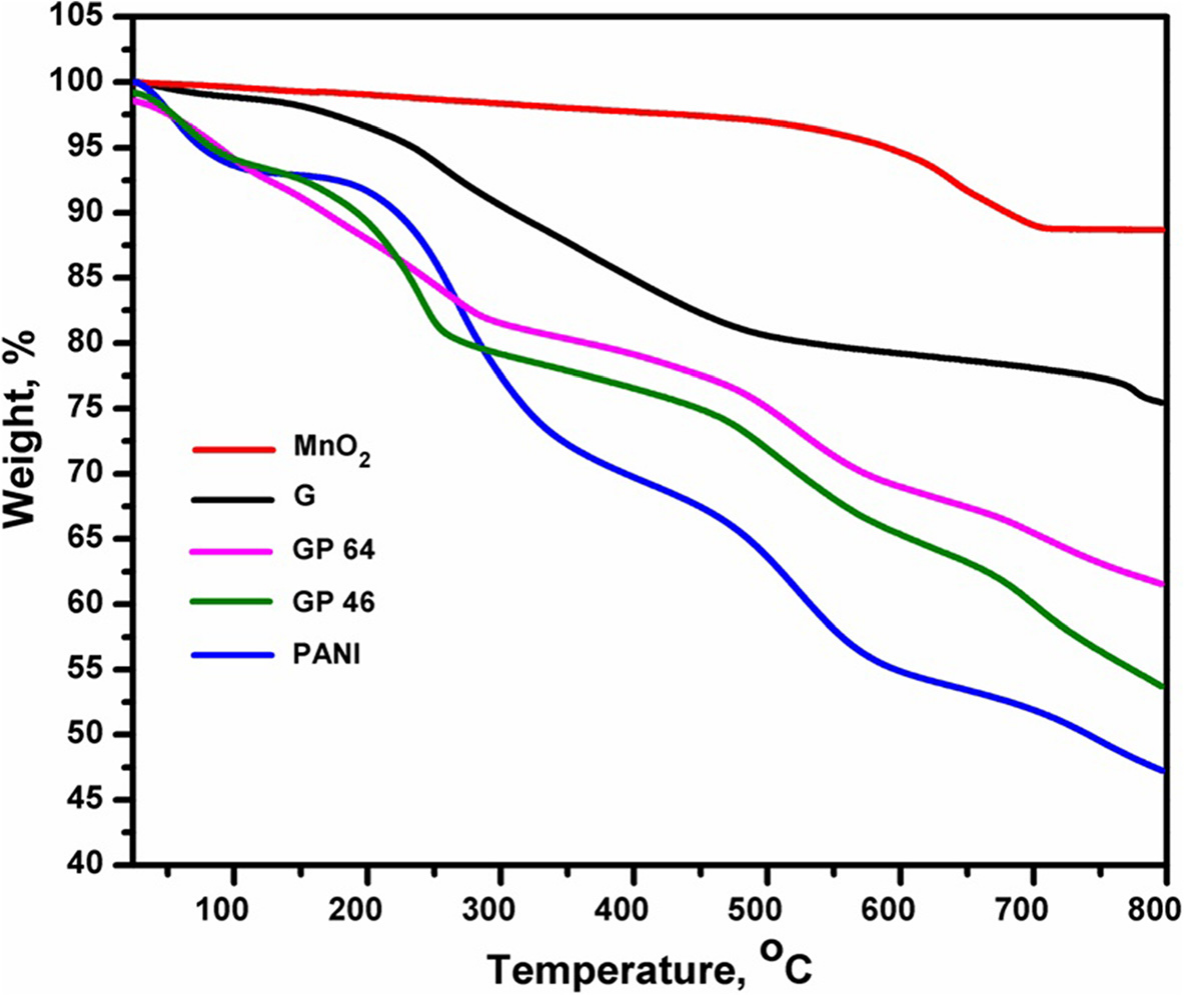
TGA of the nanocomposites. TGA of MnO_2_, G, PANI, GP 46, and GP 64 measured under the nitrogen atmosphere.

As shown in the TGA curve in Figure 3, the initial weight loss of graphene at approximately 100 C occurred because of the thermal decomposition of the residual water. The residual functional groups present in the graphene sheets deintercalated between 200 C and 300 C, and a weight loss of 25 wt.% was observed at 800 C. After the thermal decomposition of functional groups, the final residue of PANI was 47%. However, the thermal stability of PANI considerably increased with the gradual loading of graphene sheets and was attributed to the formation of G/PANI hybrid materials.

**Figure 4:**
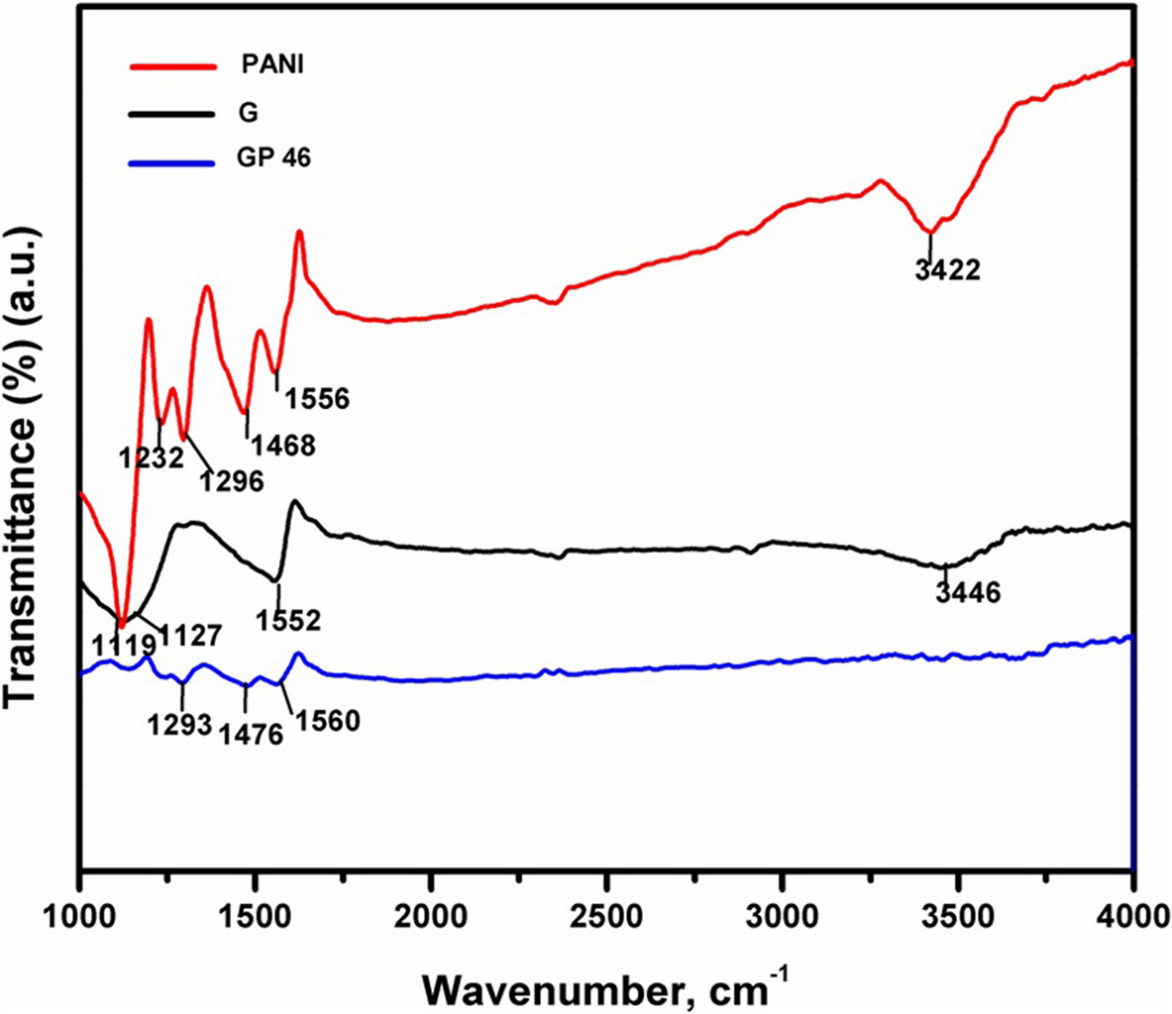
FTIR analysis of the nanocomposites. FTIR spectra of G, PANI, and GP 46 nanocomposites.

The presence of surface functional groups is commonly identified by FTIR spectroscopy. As seen in Figure 4, the absorption peaks of PANI at 1,556 and 1,468 cm^−1^ were corresponding to the C-C stretching deformation of quinoids and benzene rings, respectively, whereas, the absorption peaks for quinoids and benzene rings of the G/PANI were observed at 1,560 and 1,476 cm^−1^, respectively. The absorption peaks at 1,296 cm^−1^ of PANI and 1,293.5 cm^−1^ of the G/PANI nanocomposites are attributed to the C-N stretching band of an aromatic amine [24]. The presence of a strong band at 3,422 cm^−1^ is an indication of the N-H stretching band of an aromatic ring of the PANI. Obviously, the intensity of the peak ratio decreased from the pure PANI to the G/PANI nanocomposites, indicating the increasing stability of the nanocomposites [25]. The intense peaks observed at 3,446 and 1,552 cm^−1^ for graphene accounted for the presence of residual -OH groups and aromatic C = C stretching vibrations, respectively. The sharp peak at 3,446 cm^−1^ for graphene clearly indicates the residual -OH groups of the graphene sheets. However, the G/PANI nanocomposites showed the absence of peak at 3,446 cm^−1^, representing an electrochemical reduction of GO during the polymerization of ANI.

**Figure 5:**
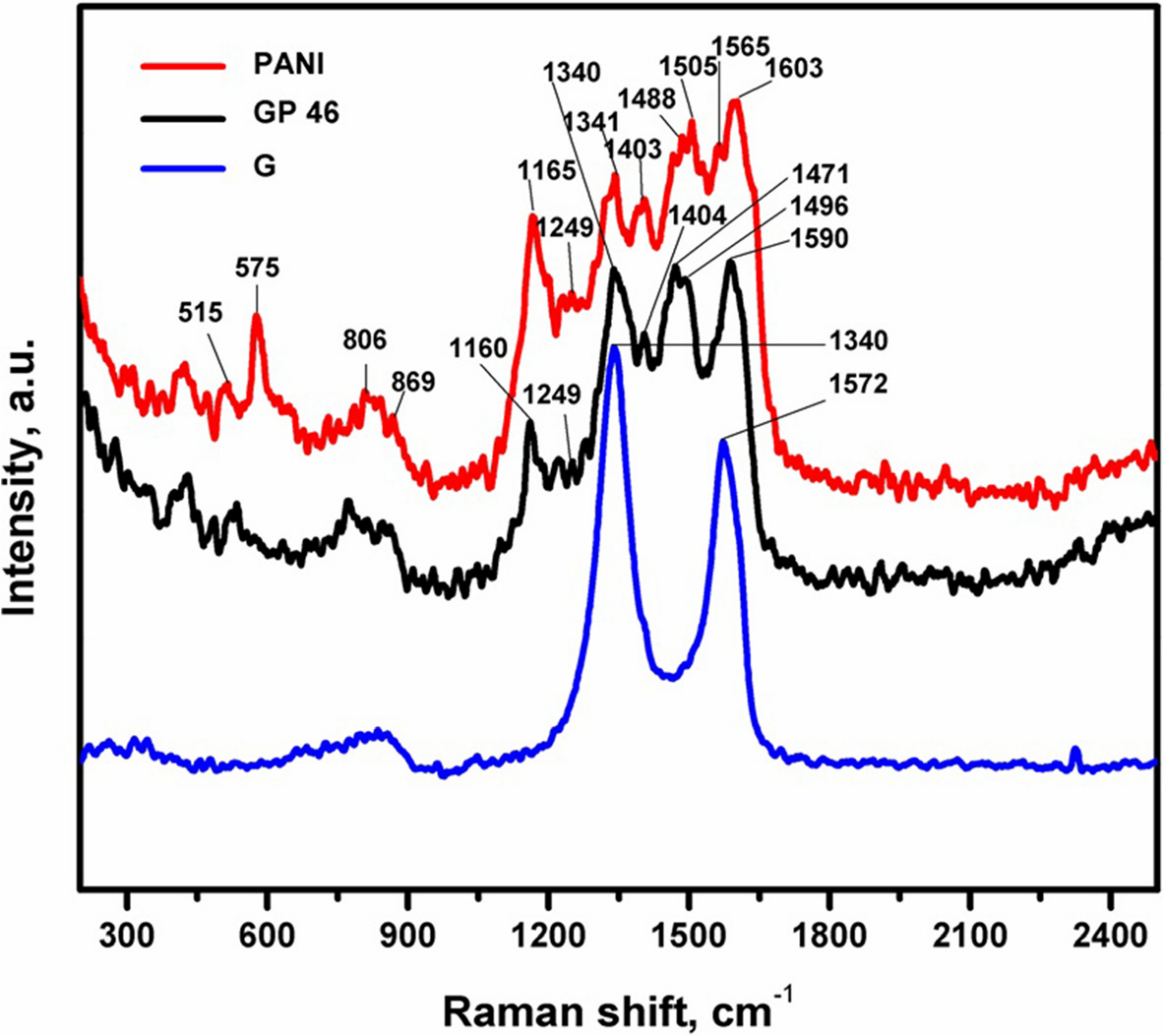
Raman spectroscopy of the nanocomposites. Raman spectra of G, PANI, GP 46 at a laser wavelength of 633 nm.

Raman spectroscopy is a powerful technique for measuring the interracial interactions between the graphene and polymers. As seen in Figure 5, the peaks observed at 1,603 and 1,505 cm^−1^ for the pure PANI correspond to the C = C stretching vibrations of benzenoid and quinoid rings, respectively, whereas the C-H bending vibrations of benzene and quinoid rings are observed at 1,249 and 1,165 cm^−1^, respectively. The reduced surfactant-treated GO shows the characteristics of *D* and *G* bands at 1,340 and 1,572 cm^−1^, respectively, corresponding to an *I*_*D*_/*I*_*G*_ ratio was 1.3. These results indicate that effective reduction of graphene oxide by hydrazine. No obvious difference was observed between the G/PANI and pure PANI, clearly indicating the formation of hybrid materials.

**Figure 6:**
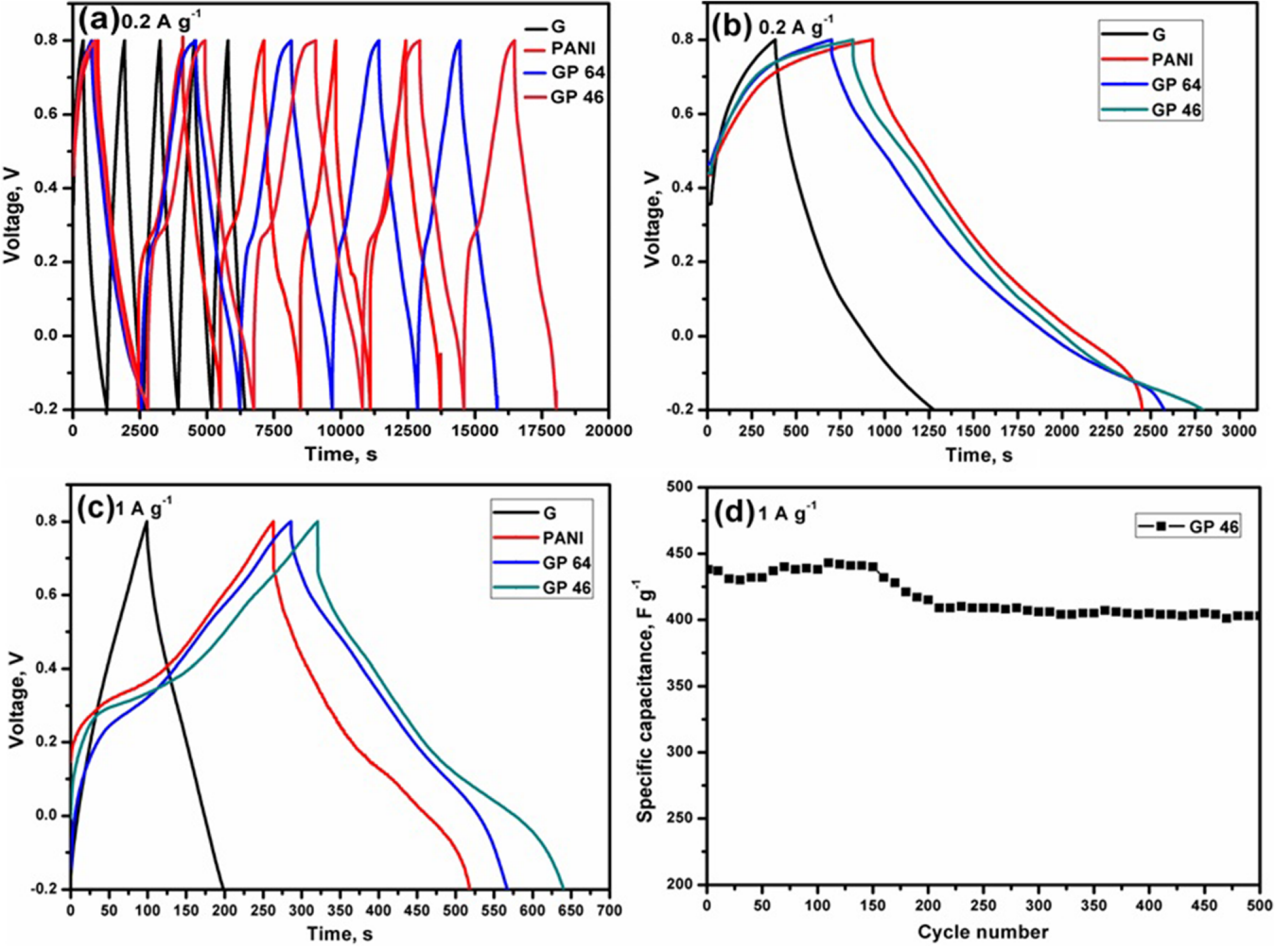
Galvanostatic charge/discharge cycles of the nanocomposites. Galvanostatic charge/discharge of **(a)** G, PANI, GP 64, and GP 46 at 0.2 A g^−1^ for the first five charge/discharge cycles. **(b)** First charge/discharge cycle of G, PANI, GP 64, and GP 46 at 0.2 A g^−1^. **(c)** First charge/discharge cycle of G, PANI, GP 64, and GP 46 at 1 A g^−1^. **(d)** Cyclic stability of GP 46 measured for 500 cycles.

The electrochemical performances of an electrode material were investigated by galvanostatic charge/discharge measurements in 2 M sulfuric acid solution. The charge/discharge of the nanocomposites was measured under the potential windows of −0.2 to 0.8 V. The specific capacitance of the G/PANI nanocomposites was investigated at different applied current densities of 0.2 and 1 A g^−1^. The specific capacitance of the G/PANI nanocomposites was estimated from the discharge cycles according to the following equation:

$$ {C}_{\mathrm{spec}}=\frac{I}{m\left(dv/dt\right)} $$

where *I* is the current loading (A), *dv/dt* is the slope of discharge curve, and *m* is the mass of the active materials (g) [5,26]. The slope of a full discharge curve (except internal resistance (IR) drop) was taken to measure the specific capacitance of the nanocomposites. Figure 6a shows the galvanostatic charge/discharge cycles of graphene, PANI, and G/PANI nanocomposites measured under the current density of 0.2 A g^−1^ for the first 5 cycles. As shown in Figure 6b, the initial specific capacitance of PANI was found to be 392 F g^−1^. The higher specific capacitance was attributed to the stronger doping effect of sulfuric acid on the PANI nanowires. The surfactant-treated graphene showed a specific capacitance of 220 F g^−1^ at 0.2 A g^−1^. Such a high specific capacitance of graphene can be attributed to the pseudocapacitive effect of the residual oxygen groups of graphene sheets [5]. Owing to the synergistic effect between the graphene and PANI, the first discharge capacitance of GP 64 and GP 46 was found to be 442 and 453 F g^−1^, respectively at 0.2 A g^−1^. Interestingly, the IR drop of the nanocomposites decreased with increasing graphene loadings, indicating the decreasing resistance of the nanocomposites electrode.

Figure 6c shows the charge/discharge characteristics of graphene, PANI, GP 64, and GP 46 at 1 A g^−1^. The discharge capacitance of GP 46 was 436 F g^−1^ at 1 A g^−1^, which is slightly lower than 0.2 A g^−1^, indicating a good synergistic effect of the PANI and surfactant-treated graphene sheets. As compared to graphene, the discharge curves of the G/PANI show the deviation from the straight line, attributed to the pseudocapacitance contribution over the electrical double-layer capacitor (EDLC) behavior. The detailed investigation of the specific capacitance of graphene, PANI, GP 64, and GP 46 is listed in Table 1. The specific capacitance of GP 46 at 1 A g^−1^ is larger than those recently reported for G/PANI nanocomposites (Table 2). The results of the higher specific capacitance of PANI and G/PANI nanocomposites at 1 A g^−1^ inferred an efficient ionic transport of PANI and G/PANI electrode during the electrochemical reactions. Figure 6d shows the electrochemical cyclic stability of GP 46. The capacitance of GP 46 was stable up to 500 cycles, and the initial capacitance retention of 91.7% was achieved at the 500th cycle. The surface loading of graphene sheets enhanced the electrical conductivity and prevented the PANI from shrinking or swelling, thus resulting in a stable cycle life.

**Table 1:** **Specific capacitance of G and G/PANI nanocomposites**

**Composites**	**Capacitance (F g** ^**−1**^ **) at 0.2 A g** ^**−1**^	**Capacitance (F g** ^**−1**^ **) at 1 A g** ^**−1**^
PANI	392	367
GP 46	453	436
GP 64	442	393
G	220	114

**Table 2:** **Specific capacitance of G/PANI and recently reported G/PANI nanocomposites at 1 A g** ^**−1**^

**G/PANI**	**Capacitance (F g** ^**−1**^ **), 3-electrode**	**Ref.**
G-PANI nanofiber	210	[1]
RGO-PANI	323	[2]
Surfactant stabilized graphene-PANI	321	[18]
G/PANI	436	Present

**Figure 7:**
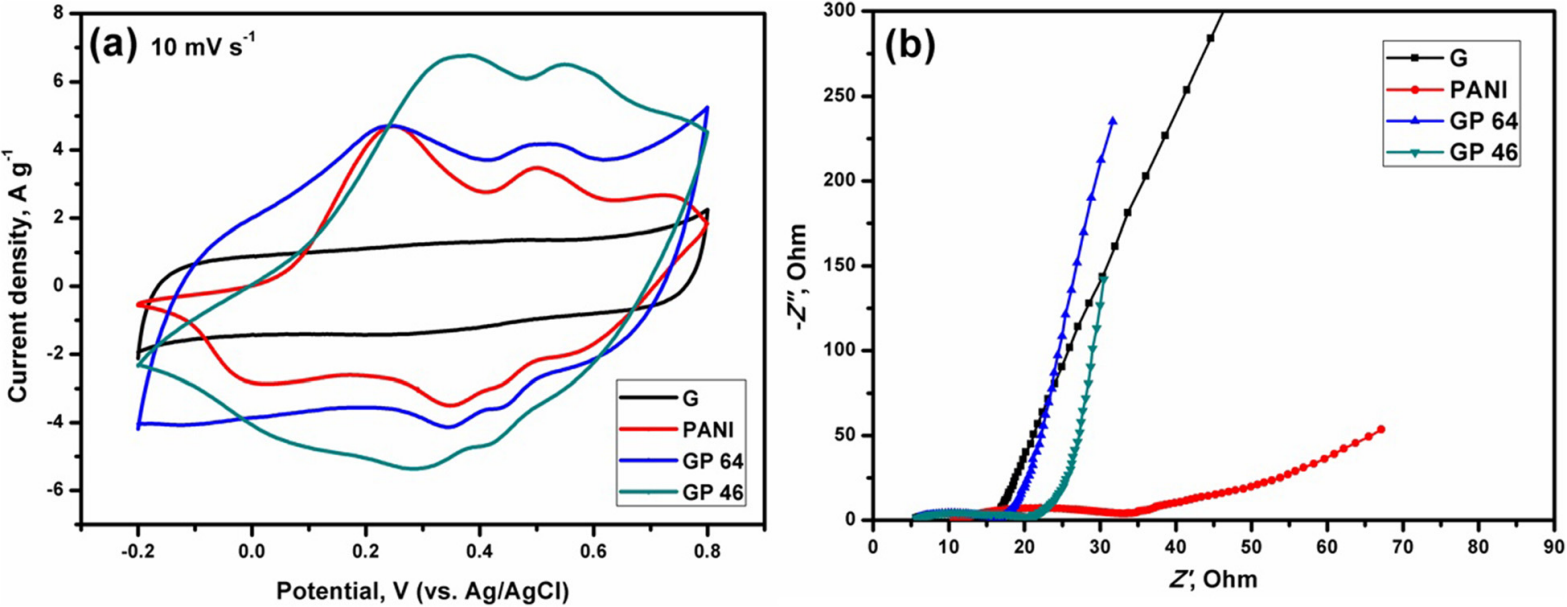
CV and EIS analysis. **(a)** CV of G, PANI, GP 64, and GP 46 nanocomposites. **(b)** EIS of the G, PANI, GP 64, and GP 46 nanocomposites.

The CV of the G, PANI, and G/PANI nanocomposites were performed at 10 mV s^−1^ (Figure 7a). The CV of G shows the complete rectangular curve, representing the characteristics of EDLC behavior [11]. However, those of G/PANI nanocomposites clearly show the rectangular peaks with redox peaks, indicating the contribution of EDLC and pseudocapacitance effect [11,24]. The increases in cyclic areas of the G/PANI nanocomposites in comparison with G and PANI are closely related to the increase of storage capacitance of the electrodes. Further, EIS analyses were conducted in order to prove the capacitance behavior and electrical resistance of the electrodes (Figure 7b). The semicircle and a straight line of the EIS at the corresponding high- and low-frequency regions indicate the charge transfer resistance and EDLC of the electrodes, respectively [1,13]. As shown in Figure 7b, the semicircles of the nanocomposites decreased with G loadings, indicating the decrease of charge transfer resistances of the electrodes. Meanwhile, the straight lines become more vertical for G/PANI nanocomposites, representing the higher EDLC behavior.

## Conclusions

The intercalation of PANI nanowires and surfactant-treated graphene allows the faster electrolyte diffusion and easy accessibility of the electrolyte at higher current densities. The electrochemical characterizations show the superior performances of GP 46 at higher current densities. For example, the specific capacitance of GP 46 at 1 A g^−1^ was as high as 436 F g^−1^ and obtained good cycle stability for 500 cycles. The excellent electrochemical performance of GP 46 at higher current density was attributed to the good synergistic effect of the PANI nanowires and graphene sheets.

## Abbreviations

ANI: aniline
CV: cyclic voltammetry
EDAX: energy-dispersive X-ray analysis
EDLC: electrical double layer capacitor
EIS: electrochemical impedance spectroscopy
FE-SEM: field emission-scanning electron microscopy
FTIR: Fourier transform infrared spectroscopy
G/PANI: graphene/polyaniline
GO: graphene oxide
GP: G/PANI
IR: internal resistance
PANI: polyaniline
SLS: sodium lauryle sulfate
TGA: thermogravimetric analysis
